# Patterns of emergency department visits during Hajj period: Towards healthcare optimization in view of Saudi Arabia's vision 2030

**DOI:** 10.12669/pjms.35.3.611

**Published:** 2019

**Authors:** Ahmad A. Mirza, Mohammed A. Alsakkaf, Amrallah A. Mohammed, Abdulrahim A. Mirza, Soha A. Elmorsy

**Affiliations:** 1 *Ahmad A. Mirza, MBBS, Department of Otolaryngology, Head and Neck Surgery, Faculty of Medicine in Rabigh, King Abdulaziz University, Jeddah, Saudi Arabia. Department of Otolaryngology, Head and Neck Surgery, Faculty of Medicine, King Abdulaziz University, Jeddah, Saudi Arabia*; 2 *Mohammed A. Alsakkaf, MBBS, Department of Surgery, Security Forces Hospital Program, Makkah, Saudi Arabia*; 3 *Amrallah A. Mohammed, MD, Department of Medical Oncology, Faculty of Medicine, Zagazig University, Zagazig, Egypt. Home Healthcare Center, King Abdullah Medical City-Holy Capital, Makkah, Saudi Arabia*; 4 *Abdulrahim A. Mirza, MBBS, Department of Surgery-Urology Section, Ministry of National Guard - Health Affairs, Jeddah, Saudi Arabia*; 5 *Soha A. Elmorsy, PhD, Department of Pharmacology, Faculty of Medicine, Cairo University, Cairo, Egypt. Department of Epidemiology and Statistics, King Abdullah Medical City-Holy Capital, Makkah, Saudi Arabia*

**Keywords:** Emergency department, Hajj, care level, patients, healthcare center, discharge, bed occupancy, inpatient care, Canadian Triage and Acuity Scale

## Abstract

**Objective:**

This study aimed to examine the pattern of emergency department (ED) visits by Hajj patients and determine the urgency of emergency visits at an advanced healthcare center.

**Methods:**

A retrospective review of medical records of Hajj patients visiting the ED at King Abdullah Medical City Makkah from September 1 to October 5, 2015 was conducted.

**Results:**

We considered 233 visits by 199 Hajj patients. Most diseases were cardiovascular related. Approximately half of the ED visits led to hospital admission, which were largely during the evening and nighttime. Potentially avoidable visits were significantly encountered during the daytime. Average bed occupation time in the ED was similar for both cases: those admitted to inpatient care and discharged from ED. Results from the Canadian Triage and Acuity Scale revealed that most patients were triaged with a score of III (48.4%) followed by a clinically better score of IV (32%); however, scores did not change significantly throughout the Hajj day.

**Conclusions:**

During Hajj, a significant proportion of patients who visited the ED at the ultimate healthcare facility were discharged within 24 hours, with a higher rate in the morning-afternoon period. Both admitted and discharged cases required equal levels of care. Therefore, an extension in working days at primary care centers and optimization of advanced healthcare facilities during Hajj is currently warranted.

## INTRODUCTION

Saudi Arabia welcomes millions of Muslims visiting the Holy Capital (Makkah or Mecca) for the annual pilgrimage of Hajj. The ritual occurs from 8 to 13 Dhul Hijjah, the last month of the Islamic year. Mass gathering and overcrowded movement from one place to another, which makes the Hajj period distinguishable, increase the risk of communicable and noncommunicable medical conditions. Additionally, the emergency departments (EDs) receive numerous cases involving acute conditions during Hajj, resulting in significantly high rates of morbidity and mortality.^1^,^2^

Different communicable and noncommunicable medical conditions, including cardiovascular events, respiratory diseases, heat stroke, and trauma, have been reported among Hajj pilgrims presenting at primary and secondary medical centers.^3^-^5^ Besides, cardiac conditions are common and urgent entities provisionally diagnosed at advanced care facilities.^2^,^6^

Nonurgent hospital visits have become an increasing concern in Middle Eastern countries, in which up to 88.7% patients at EDs were labeled nonurgent.^7^,^8^ In Saudi Arabia, two studies have examined the extent of these potentially avoidable visits in non-Hajj periods, and found that 53% to 59.4% patients visiting EDs presented with primary care or nonurgent conditions.^7^,^9^

The Saudi Ministry of Health provides high-quality, free health services to all Hajj pilgrims. Therefore, the Saudi authorities assigned 25,000 health personnel and allocated 25 hospitals with 155 permanent and seasonal health centers, devoted to providing different levels of medical care, during Hajj. These services are distributed across all four major ritual localities: Mina, Arafat, and the two Holy Mosques. Approximately 60% patients admitted to some of these care centers are transferred to advanced care facilities.^10^ King Abdullah Medical City-Holy Capital (KAMC-HC) hospital is a recently established ultimate and quaternary care hospital in the region that deals with complicated cases, providing advanced healthcare for all pilgrims visiting the center with its 24-hour emergency services.

Previous studies on Hajj documented the pattern of admission among Hajj pilgrims, showing different diagnoses in this group.^2^,^6^,^10^,^11^ However, no previous studies have elucidated the pattern of acute cases treated at the ultimate healthcare center during Hajj and none described the urgency of emergency visits during this vital period.

Therefore, we aimed to examine the pattern of ED visits (EDVs) among Hajj patients and determine the urgency of EDVs during Hajj. This would ultimately help to maintain a high level of preparedness for Hajj-related potential disasters and optimize the large healthcare facilities during Hajj.

## METHODS

The study was conducted at the KAMC-HC after approval from its Institutional Review Board. Retrospective data analyses were conducted using medical records of Hajj patients admitted to the ED between September 1 and October 5, 2015.

During Hajj, patients who sought care at the KAMC-HC hospital were triaged to the outpatient department for further medical care or treated at the ED. From the ED, they were discharged (these visits were considered potentially avoidable), admitted to an inpatient unit after receiving initial treatment, were deemed absconding or declared dead.

Data on demographic details, time of presentation at the ED, admission type (primary or referral), clinical diagnosis and comorbidities, disposition and time of disposition, language barriers, and Canadian Triage and Acuity Scale (CTAS) score classification^12^ were collected from patients’ medical records. Type of admission was classified as “primary” if the patients presented directly to the ED, and “referral” if they had been evaluated in other care centers and subsequently referred to ours. Bed occupation duration was calculated for both admitted and discharged patients as the period from the start of the EDV to hospital admission or discharge. CTAS helps healthcare professional to determine the severity of cases at the triage stage. The scores range from I (the need for resuscitation) to V (nonurgent). EDV times were classified into three equal periods: A (08:00 a.m. to 03:59 p.m.), B (04:00 p.m. to 11:59 p.m.), and C (12:00 a.m. to 07:59 a.m.).

### Statistical Analysis

Data were analyzed using SPSS version 21.0. Numeric data were presented as means and standard deviations or medians and interquartile ranges. Comparisons were made using ANOVA, Mann-Whitney U tests, or Kruskal-Wallis tests according to data distribution. The categorical variables were compared using chi-square tests. The bed occupation duration was compared between patients admitted to the hospital and those discharged from the ED. Comparisons were also made based on CTAS scores. Spearman’s rank correlation coefficient was used to assess the relationship between CTAS score and EDV duration.

## RESULTS

**Fig.1:**
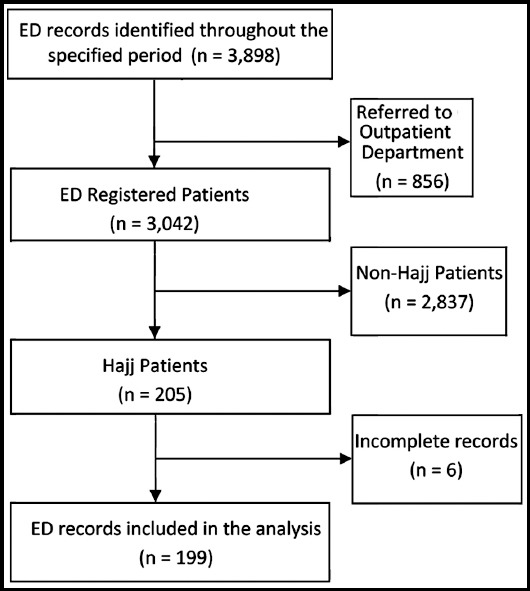
Flowchart of emergency department visits during Hajj. (ED: Emergency Department).

In total, 3,898 records of patients who visited the ED during the study period were assessed. Of these, 3,042 (78.0%) were registered and evaluated in the ED. In total, 205 (6.7%) were Hajj patients. Six (2.9%) records were excluded because of insufficient data (Fig.1).

Of the 199 patient records included in the analysis, 64.3% (n = 128) were of men with a mean age of 60.6 ± 16.5 years, and 4% (n = 8) patients reported the need for a translator. The total number of EDVs was 233, with mean 1.17 ± 0.5 (median= 1, range: 1 - 4), including 174 (87.4%) patients who visited once, 23 (11.6%) who visited two to three times, two (1%) who visited more than three times. A total of 114 (85.7%) visits were direct (primary) visits, while 19 (14.3%) visits were referrals.

The most common provisional diagnoses were cardiovascular-related diseases (33.9%), heat stroke (21.9%), and respiratory diseases (14.6%). ED records also showed that the most common comorbid conditions were hypertension (45.2%), diabetes mellitus (41.1%), and ischemic heart disease (23.2%; Table-I).

**Table-I:** Provisional diagnoses and recorded comorbidities of 199 Hajj patients admitted to the emergency department.

Variable	n	%
***Provisional diagnosis***		
Cardiovascular-related diseases	79	33.9
Heat stroke	51	21.9
Respiratory diseases	34	14.6
Neurological diseases	22	9.4
Trauma	20	8.6
Metabolic-related, electrolyte disturbance, dehydration	17	7.3
Unspecified pain for further investigation	11	4.7
Fever for further investigation	4	1.7
Others	30	12.9
***Comorbidities***		
Hypertension	76	45.2
Diabetes mellitus	69	41.1
Ischemic heart disease	39	23.2
Others	38	23.0

Visiting times, durations, and outcomes for 233 EDVs are shown in Table-II. Most EDVs were encountered before midnight (Periods A and B; 89.7%). Approximately half of EDVs (57.1%) required admission, and the highest proportion of discharged visits occurred during Period A (61.6%). Furthermore, admission occurred more frequently during Periods B and C (70.1% and 78.3%, respectively) than Period A (38.4%; p < 0.001).

**Table-II:** Characteristics of 233 emergency department visits (EDVs) of 199 Hajj patients.

Variable	n	%
***EDV Period***		
A (08:00-16:00)	112	48.1
B (16:00-00:00)	97	41.6
C (00:00-08:00)	24	10.3
***Bed Occupation Duration (hours)^†^***		
≤1	52	27.7
>1 to 2	33	17.6
>2 to 4	54	28.7
>4 to 5	24	12.8
>5	25	13.3
***ER Outcome***		
Admission	133	57.1
Regular ward	67	50.4
Intensive care unit	66	49.6
ED Mortality	none	0
Discharge	100	42.9
Discharge home	96	96
Discharge against medical advice	4	4

† The time was not calculated in 11 EDVs. ER: Emergency Room; ED:

Emergency Department; EDV: Emergency Department Visit

The average bed occupation duration was 3.0 ± 2.9 h. However, when bed occupation durations for admitted cases were compared with those for discharged (potentially avoidable) cases, no statistically significant difference was observed (p = 0.24). In more than half the patients, CTAS scores were III or lower. There was no significant correlation between CTAS score and EDV duration (*R* = -0.136, p = 0.184), neither when it was compared across daily periods (p = 0.59; Table-III).

**Table-III:** Differences by time of emergency department visit.

Variable	Shift A No (%)	Shift B No (%)	Shift C No (%)	P^†^value
Hospitalization	43(38.4)	68(70.1)	18(78.3)	<0.001
Discharge	69(61.6)	29(29.9)	5(21.7)
***CTAS Score^‡^***				
I	1 (1.4)	0 (0)	0 (0)	0.587
II	6 (8.7)	5 (12.8)	4 (28.6)	
III	35 (50.7)	18 (46.2)	6 (42.9)	
IV	21 (30.4)	14 (35.9)	4 (28.6)	
V	6 (8.7)	2 (5.1)	0 (0)	

† P value <0.05 was considered significant.

‡ Available only for 122 EDVs.

CTAS: Canadian Triage and Acuity Scale

## DISCUSSION

This study examined the pattern of Hajj patients’ EDVs and the urgency of these visits. The distribution of age and sex in this study was similar to that documented in other studies exploring Hajj.^2^,^3^ Elderly male Hajjis were predominant in the study. Most pilgrims had saved their income for decades to perform this Islamic ritual. Previous studies examining disease patterns during the Hajj revealed prevalent noncommunicable diseases and chronic diseases in elderly people. Most published studies on Hajj reported that respiratory, cardiac, neurological, and gastrointestinal diseases were common in pilgrims admitted to hospitals for medical care during the Hajj.^2^,^3^,^10^,^11^,^13^ Moreover, most studies showed that respiratory diseases were the leading cause of admission during Hajj,^3^,^10^,^11^,^13^ while in the current study, it was the third most common cause of EDVs. Cardiovascular disease was the primary cause of EDVs in the current study, accounting for approximately one-third of all patients. Khan et al.^2^ reported a similar high rate of cardiovascular disease (i.e., 34%). Moreover, in Al-Mashaer centers, common comorbidities reported for admitted patients during Hajj included asthma and chronic obstructive pulmonary disease (22.5%), hypertension (17.5%), and diabetes mellitus (15%).^10^

Notably, most studies reported mortality rates ranging from 0.6%^11^ to 16%,^2^ while we did not observe any deaths. This could be partially attributed to the highly developed and adequately equipped ED at the KAMC-HC hospital; however, we did not consider mortality data after admission to in-patient care units. Appropriate screening of prospective pilgrims for cardiovascular risk factors and adjustment to their treatment during the Hajj has decreased morbidity and mortality in them.^14^ However, we are not aware of any recent changes in the screening methods that could have influenced morbidity and mortality in this cohort. Additionally, we have no reported cases of meningitis. This mostly due to the strict regulation of vaccines received by pilgrims who intended to visit Makkah during Hajj.

Most patients in our study were seen at the ED during the day or evening (Periods A and B), which coincided with most Hajj activities. Nevertheless, hospital admission occurred mainly for those who presented to the ED during the evening or late night (Periods B and C). This could be attributed to the fact that significant emergency illnesses usually appear after the crowded mass movement. In particular, during Mina rituals, many pilgrim groups walk toward Jamarat concomitantly within the hours before sunset, which corresponds with the beginning of Period B. In fact, the disastrous Hajj stampede at Mina in 2015, which coincided with the study, occurred at this time of day (Period B). Additionally, this particular period succeeds a period involving an inappropriate pattern of EDVs (Period A), when the highest proportion of potentially avoidable visits occurred. Consequently, full occupancy of emergency beds and sub-optimization of healthcare facilities and resources might be witnessed at the beginning of this critical period (Period B). Despite the variation in the visits pattern, there was no remarkable difference in CTAS scores between the periods.

The average duration of EDVs was three hours, which is well within the international standard.^15^-^16^ However, studies in EDs in the UK showed variation in the durations of visits according to admission hour and patient characteristics.^16^

Approximately half of the patients treated in the ED were discharged directly and not admitted for further treatment or referred to other hospitals. It is noteworthy that the Saudi government honorarily provides high-quality care and easy access to hospitals to all Hajj pilgrims including those with less severe conditions. The extent to which patients with non-serious conditions were presented to the ED and their contribution to the delay in patient flow remain controversial. Some studies conducted in the USA and Canada showed effects of these factors on waiting times,^17^-^19^ whereas others showed only negligible effects on waiting times for other ED patients.^20^ Additionally, the current study showed similar bed occupation durations for both types of patients (i.e., those who were admitted and discharged). This equality might be unfavorable for the former.

Inappropriate use of ED facilities is common, and has a range of reasons, but patient preference appears to be the most common factor.^18^ It is widely understood that avoidable EDVs contribute to higher healthcare costs and lead to poor cost efficiency in healthcare delivery.^21^ This should be considered as Saudi Arabia has recently launched a promising national vision (“KSA Vison 2030”), which strives to establish a thriving economy and fulfilling lives for citizens, while promoting physical well-being.^22^ However, some studies conducted in the USA report only minor savings when non-severe cases could be referred elsewhere.^23^,^24^ Nevertheless, healthcare delivery at appropriate levels leads to more efficient use of healthcare services and improved patient outcomes. Healthcare provided in EDs cost more than those in alternative primary care settings;^21^ therefore, optimal usage of resources is warranted.

From patients’ perspective, it remains unclear whether avoidable ED cases would receive more appropriate care elsewhere. However, primary or secondary care facilities are considered the most equipped for such cases.

### Limitations

One limitation of the study was the lack of comparison with non-Hajj patients during the same period. Potential differences in health-seeking behavior between these two groups could clarify whether avoidable visits are a Hajj-specific or general problem. Therefore, further exploration needs to identify suitable ways for advanced healthcare centers to concentrate primarily on severe and urgent cases and provide cost-efficient services.

## CONCLUSION

During Hajj, approximately half of the patients who visited the ED were discharged. Most of these potentially avoidable visits encountered in the morning-afternoon. Bed occupancy durations in the ED did not differ between potentially avoidable cases and those that necessitated further treatment. An extension in working days at primary healthcare centers and optimization of large facilities is currently warranted to provide an optimal care for pilgrims during Hajj.
